# Elevated ACE Levels Indicate Diabetic Nephropathy Progression or Companied Retina Impaired

**DOI:** 10.3389/fcdhc.2022.831128

**Published:** 2022-05-12

**Authors:** Kangkang Huang, Yunlai Liang, Kun Wang, Yating Ma, Jiahui Wu, Huidan Luo, Bin Yi

**Affiliations:** ^1^Department of Clinical Laboratory, Xiangya Hospital, Central South University, Changsha, China; ^2^National Clinical Research Center for Geriatric Disorders, Xiangya Hospital, Central South University, Changsha, China

**Keywords:** type 2 diabetic mellitus, diabetic nephropathy, angiotensin converting enzyme, diabetic retinopathy, RAAS

## Abstract

**Objectives:**

Renin-angiotensin-aldosterone system plays important roles in the development of diabetic nephropathy (DN), and angiotensin converting enzyme (ACE) is the key factor in the process from angiotensin I to angiotensin II, but the variation and roles of serum ACE in DN patients are still unclear.

**Methods:**

Forty-four type 2 diabetes mellitus (T2DM) patients, 75 DN patients, and 36 age-gender-matched healthy volunteers were recruited who attended Xiangya Hospital of Central South University in this case control study. Serum ACE levels and other indexes were tested with commercial kit.

**Results:**

ACE levels in DN were significantly higher than T2DM and controls (F = 9.66, *P* < 0.001). Serum ACE levels significantly correlated with UmALB (r = 0.3650, *P* < 0.001), BUN (r = 0.3102, *P* < 0.001), HbA1c (r = 0.2046, *P* = 0.0221), ACR (r = 0.4187, *P* < 0.001), ALB (r = -0.1885, *P* = 0.0192), and eGFR (r = -0.3955, P < 0.001), and we got an equation that Y = 2.839 + 0.648X_1_ + 2.001X_2_ + 0.003X_3_ - 6.637X_4_ +0.416X_5_ - 0.134X_6_ (Y: ACE; X_1_: BUN; X_2_: HbA1C; X_3_: UmALB; X_4_: gender; X_5_: ALB; X_6_: eGFR, R^2^ = 0.655). When DN patients were divided into advanced-stage and early-stage with or without DR, ACE levels would increase when early-stage DN develops into advanced-stage or companied with DR.

**Conclusion:**

Elevated serum ACE levels may hint DN progression or retina impaired of DN patients.

## Introduction

Diabetes mellitus is a kind of metabolic disease with abnormal higher blood glucose. It was estimated that 463 million people were diagnosed with DM in 2019, and this number will arise to 700 million by 2045 among aged 18–99 years (1). Long term hyperglycemia causes general vascular damage of kidneys, eyes, nerves, and heart and leads to microvascular complications (2), such as diabetic nephropathy (DN), diabetic retinopathy (DR), and diabetic peripheral neuropathy. Approximately 25–35% (3) of DM patients will develop into DN, which is one of the leading pathological causes of end-stage renal disease worldwide and is the most common cause of nephropathies requiring renal replacement therapy in many nations (4).

Except renal replacement therapy, researchers reported potential drugs such as empagliflozin (5), sirtuins 3 (SIRT3) (6), linagliptin (7), rho-associated kinase (ROCK) inhibitors (8), mineralocorticoid receptor antagonist (9), and peptide N-acetyl-seryl-aspartyl-lysyl-proline (AcSDKP) (10) could reduce the progression of DN in diabetic patients. The most common DN treatments are based on the RAAS system inactivation, precisely with the use of either the ACE inhibitors (ACEis) or angiotensin receptor blockers (ARBs) or their combination; however, ACEi could elevate AcSDKP level, whereas ARB does not (11). Physiologically, renal epithelial cells are associated tightly with their neighbors, which prevent their potential for movement and dissociation from the epithelial layer. Under the effect of high glucose concentrations, glomerular podocyte would appear the phenotypic change of epithelial-to-mesenchymal transition (EMT) and endothelial to mesenchymal transition (EndMT) (12, 13). When EMT and EndMT occurred, endothelial cells lost their typical phenotype to acquire mesenchymal features, characterized by the development of invasive and migratory abilities as well as the expression of typical mesenchymal products such as α-smooth muscle actin and type I collagen (12). The glucocorticoid receptor (GR) is a nuclear hormone receptor that is expressed ubiquitously in most cell types. A previous study has reported that loss of endothelial GR activates Wnt signaling pathway. This pathway is known to be upregulated in renal fibrosis (14). The most important inducer of kidney fibrosis is TGF-β, which could trigger EndMT by activating specific AKT and Smad signaling pathways (15). Fibroblast growth factor 1 (FGFR1) as mitogen and insulin sensitizer could suppress inflammation and renal glomerular and tubular damage through inhibiting the activation of nuclear factor κB and c-Jun N-terminal kinase signaling pathways (16). Notch signaling and Hedgehog signaling are also involved in the development of DN, Notch signaling promotes diabetic glomerulopathy (17) and tubulointerstitial fibrosis (18) and inhibits endothelial cell proliferation and migration (19), and Hedgehog signaling could reduce reactive oxygen species production through increasing superoxide dismutase and catalase production (20–22).

Several factors were proved associated with the occurrence of DN. DN is a prototype disease of the activation of renin-angiotensin-aldosterone system (RAAS) (23), including angiotensinogen, angiotensin receptor, Ang I, Ang II, ACE, and renin. Angiotensinogen is converted to Ang I by renin and Ang I transformed into Ang II through ACE. ACE gene polymorphisms have implications in the pathophysiology of diabetes developing into DN. It has been reported in several studies that D allele is a risk factor for DN and I allele is a protective factor for DN in Asian people (24, 25). Further study implicated that D/D genotype is an independent risk factor for the development of DN in Chinese population with T2DM (25, 26). In adults, plasma ACE level does not change with age, but it is affected by factors like environment or lifestyle (26, 27). Plasma ACE level in I/D and D/D genotype people was reported to be 30% and 60% higher, respectively, than that in I/I genotype people (27).

Hence, what the variation of plasma ACE level in early and advanced stage DN patients with or without DR attracts us. In this research, we elaborated that serum ACE levels elevated in DN patients compared with T2DM and healthy. In the subgroup analysis, we separated DN patients with urinary microalbumin creatinine ratio (ACR) at the cut-off of 300 mg/g to early and advanced stage; meanwhile, we consider the DN patients whether retinal damage. As for DN patients with uninjured retina, increased serum ACE levels hint that early stage developed into advanced stage, and serum ACE levels raised are also a reminder of retina impaired in early-stage DN patients. The results of our study make a point that serum elevated ACE levels are a marker for progression of DN and early-stage DN patients with impaired retina.

## Materials and Methods

### Participants

A total of 155 participants were recruited in this study from August 2018 to March 2020 in Xiangya Hospital of Central South University. Forty-four T2DM patients averaged 57.82 ± 9.56 years meeting the American Diabetes Association (ADA) standard in 2018 (28), including fasting blood-glucose (FPG) level of ≥126 mg/dl (7.0 mmol/L), a 2-h plasma glucose (2h-PG) level of ≥200 mg/dl (11.1 mmol/L) during OGTT, a hemoglobin A1c (HbA1c) level of ≥6.5% (48 mmol/mol), or a random plasma glucose ≥200 mg/dl (11.1 mmol/L) in a patient with classic symptoms of hyperglycemic crisis. Seventy-five patients averaged 56.60 ± 13.45 years were defined as DN on the condition of their ACR > 30 mg/g or eGFR < 60 ml•min^-1^•1.73 m^2^ (29). Patients with type 1 diabetes mellitus (T1DM) or other kidney disease (like nephropathy syndrome or nephritis), cancer, cardiovascular disease, severe lung or liver disease, or infectious disease, or treated with nephrotoxic drugs or other drugs that could influence urinary albumin excretion, or receiving medications of angiotensin receptor blocker (ARB) or angiotensin-converting enzyme inhibitor (ACEI) were excluded. Meanwhile, 36 (53.33 ± 7.14 years) age-gender-matched healthy volunteers were introduced in this study. DR diagnosis was made by an ophthalmologist through direct ophthalmoscopy with retinal vascular structural changes such as microaneurysm, intraretinal hemorrhage, vascular circuity, and vascular malformation (30). Participants in this study were given informed consent, and this study was permitted by the ethics committee of Xiangya Hospital of Central South University (No 202009119).

### Samples

Venous blood was collected after a minimum of 8 h fasting diet and then centrifuged at 3600 rpm for 5 min. The isolated serum samples were frozen at -20°C until test.

### Clinical and Laboratory Indexes

Clinical parameters of each participants were obtained including diagnostic message, gender, age, BMI, and laboratory indexes performed on blood samples containing ALB (albumin), TG (total triglycerides), TC (total cholesterol), HDL-C (high density lipoprotein cholesterol), LDL-C (low density lipoprotein cholesterol), TB (total bilirubin), DB (direct bilirubin), TBA (total bile acid), BUN (blood urea nitrogen), Scr (serum creatinine), UmALB (urine microalbumin), Ucr (urine creatinine), and HbA1c (glycosylated hemoglobin) measured on AU5800 automatic analyzer (Beckman Coulter, CA, USA). Serum ACE levels were tested with appropriate Commercial kit (DEROM Biomedical Engineering Co., LTD, Hunan, China). In addition, eGFR (estimated glomerular filtration rate) was computed by modified MDRD equation and ACR (urinary microalbumin creatinine ratio) was also calculated as below: eGFR [ml.min^-1.^ (1.73m^2^)] = 175_*_[Scr (mg/dl)]^-1.154^_*_ (age)^-0.203^, female multiple with 0.742, ACR (mg/g) = UmALB _*_ (113.1_*_ Ucr) ^-1^_*_10^6^.

### Statistical Analysis

Statistical analyses were performed using SPSS (Version 26, SPSS Inc., Chicago, IL, USA). The normal distribution quantitative statistics were presented as mean ± SD, the differences among groups were compared with ANOVA, and SNK test was used to verify the differences between two groups. Gender as dichotomous data was coded as “male=1” and “female=0”, and the difference among groups used chi-square test. Pearson correlation coefficients were reported for correlations between ACE and other indexes. Independent variable potentially influencing plasma ACE levels was tested by multiple linear regression analysis with input α = 0.05, output α = 0.1. All the tests were two-tailed, and *P* < 0.05 was considered statistically significant.

## Results

### Patient Baseline Characteristics

Baseline characteristics of the participants in our research were summarized in **Table 1**. ACE levels were 19.46 ± 7.67 (μmol/L) in the control group, 18.75 ± 12.96 (μmol/L) in T2DM, and 27.12 ± 11.93 (μmol/L) in DN, respectively. As shown in **Supplementary Figure S1**, the ACE levels in the DN group were remarkably elevated compared to that in the control and in T2DM (*P* < 0.01), whereas there was no significant difference of ACE levels between the T2DM group and the control. Meanwhile, the differences of age, BMI, duration, TBA, LDL, TC, and HDL/LDL between groups were not significant. Both the levels of BUN and Scr in DN patients were significantly higher than that in the control or T2DM patients, while the concentration of ALB, eGFR, TB, and DB in DN group was distinctly declined in contrast to the control or T2DM patients. HbA1c levels in DN or T2DM group were greatly higher than that in the control group.

**Table 1:** Characteristics of the subjects included in this study (mean ± SD).

Parameter	Control	T2DM	DN	F	*P* value (two-tailed)
Age (years)	53.33 ± 7.14	57.82 ± 9.56	59.75 ± 9.02	2.06	0.13
Gender, M/F, n	20/16	26/18	50/25	0.001	0.973^*^
BMI (kg/m^2^)	21.51 ± 2.98	22.44 ± 2.53	25.64 ± 23.22	0.99	0.376
ACE (μmol/L)	19.46 ± 7.68	18.75 ± 12.96	27.12 ± 11.93	9.66	0.000
ALB (g/L)	46.34 ± 3.38	39.64 ± 5.87	33.96 ± 7.61	47.07	0.000
TG (mmol/L)	1.47 ± 0.75	2.02 ± 1.58	2.70 ± 3.24	3.19	0.044
TC (mmol/L)	4.93 ± 0.88	4.34 ± 1.25	4.89 ± 1.86	2.16	0.119
HDL (mmol/L)	1.38 ± 0.36	1.05 ± 0.48	1.16 ± 0.83	2.55	0.082
LDL (mmol/L)	3.02 ± 0.68	2.74 ± 0.87	3.06 ± 1.24	1.40	0.25
HDL/LDL.	0.45 ± 0.19	0.47 ± 0.54	0.48 ± 0.74	0.02	0.978
TB (μmol/L)	10.98 ± 3.98	10.63 ± 4.53	7.98 ± 4.28	8.36	0.000
DB (μmol/L)	5.49 ± 1.98	4.68 ± 1.85	3.70 ± 2.05	10.57	0.000
TBA (μmol/L)	3.58 ± 2.18	6.65 ± 6.31	6.59 ± 8.27	2.78	0.065
UmALB (mg/L)	/	9.80 ± 7.78	970.91 ± 156.49	24.69	0.000
BUN (μmol/L)	4.59 ± 1.55	5.41 ± 1.89	9.19 ± 6.26	16.51	0.000
Scr (μmol/L)	74.33 ± 19.42	79.44 ± 18.97	230.46 ± 405.61	5.67	0.004
HbA1c (%)	5.36 ± 0.33	8.27 ± 3.07	8.07 ± 2.26	10.34	0.000
UCr (mol/L)	/	6704.52 ± 3713.47	5835.59 ± 3764.71	5.86	0.017
eGFR [ml.min-1. (1.73m2)]	82.96 ± 18.19	81.18 ± 17.97	48.99 ± 31.33	32.63	0.000
ACR (mg/g)	/	13.83 ± 7.74	2090.25 ± 2884.91	22.72	0.000

^*^Chi-square test and P < 0.05 was considered statistically significant.

### Correlations Between ACE and Other Indexes

We used scatter diagram to explore the correlations between ACE and other laboratory indexes such as ALB, UmALB, BUN, Scr, GFR, BMI, TB, DB, TBA, HbA1c, and ACR. As a result, we found that ACE level significantly correlated with UmALB (r = 0.3650, *P* < 0.001), BUN (r = 0.3102, *P* < 0.001), HbA1c (r = 0.2046, *P* = 0.0221), ACR (r = 0.4187, *P* < 0.001), ALB (r = -0.1885, *P* = 0.0192), and eGFR (r = -0.3955, *P* < 0.001) as **Table 2** described. Specifically, it had closely positive correlations with UmALB (r = 0.4418, *P* < 0.0001), BUN (r = 0.3082, *P* < 0.0001), HbA1c (r = 0.2227, *P* = 0.0129), or ACR (r = 0.4094, *P* < 0.0001), and negative correlations with ALB (r = -0.1885, *P* = 0.0192) or eGFR (r = -0.4091, *P* < 0.0001) as shown in **Supplementary Figure S2**, but no other significant correlations were observed between ACE levels and other laboratory data.

**Table 2:** Correlation between ACE and some indexes.

Parameter	UmALB	BUN	HbA1c	ACR	ALB	eGFR
r	0.3650	0.3102	0.2046	0.4187	-0.1885	-0.3955
R^2^	0.1332	0.0962	0.0419	0.1753	0.0355	0.1564
95% CI	(0.1917–0.5163)	(0.1604–0.4460)	(0.0301–0.3671)	(0.2519–0.5613)	(0.3365, 0.03124)	(-0.5207–0.2537)
*P* value (two-tailed)	<0.001	<0.001	0.0221	<0.001	0.0192	<0.001

### The Influence Factors of ACE

We used multiple logistic regression to investigate the influence factors of serum ACE level in serum. ACE level was defined as the dependent variable, and predictor variables include BUN, HbA1c, UmALB, gender, ALB, eGFR, BMI, input α = 0.05, and output α = 0.1. The equation was achieved as follows: Y = 2.839 + 0.648X_1_ + 2.001X_2_ + 0.003X_3_ - 6.637X_4_ + 0.416X_5_ - 0.134X_6_ (Y: ACE, X_1_: BUN, *P* = 0.093; X_2_: HbA1c, *P* = 0.000; X_3_: UmALB, *P* = 0.010; X_4_: gender, *P* = 0.006; X_5_: ALB, *P* = 0.014; X_6_: eGFR, *P* = 0.031, R^2^ = 0.655). The results given in **Table 3** presented that these variables could influence 65.5% of the ACE levels in the serum and only 35.5% of the ACE affected by accidentia or other factors. The standardized regression coefficients of X_1_-X_6_ were 0.197, 0.387, 0.267, -0.227, 0.232, and -0.279, respectively, implicating that HbA1c could affect the ACE level most.

**Table 3:** The influence factors of ACE established by multiple logistic regression model.

Parameter	BUN	HbA1c	UmALB	Gender	ALB	eGFR	Constant
β	0.648	2.001	0.003	-6.367	0.416	-0.134	2.839
β**^’^**	0.197	0.387	0.267	-0.227	0.232	-0.279	
t	1.697	4.722	2.621	-2.789	2.497	-2.196	
*P* value (two-tailed)	0.093	0.000	0.010	0.006	0.014	0.031	

### ACE Level in Patients With DN and DN Combined With DR

**Figure 1:**
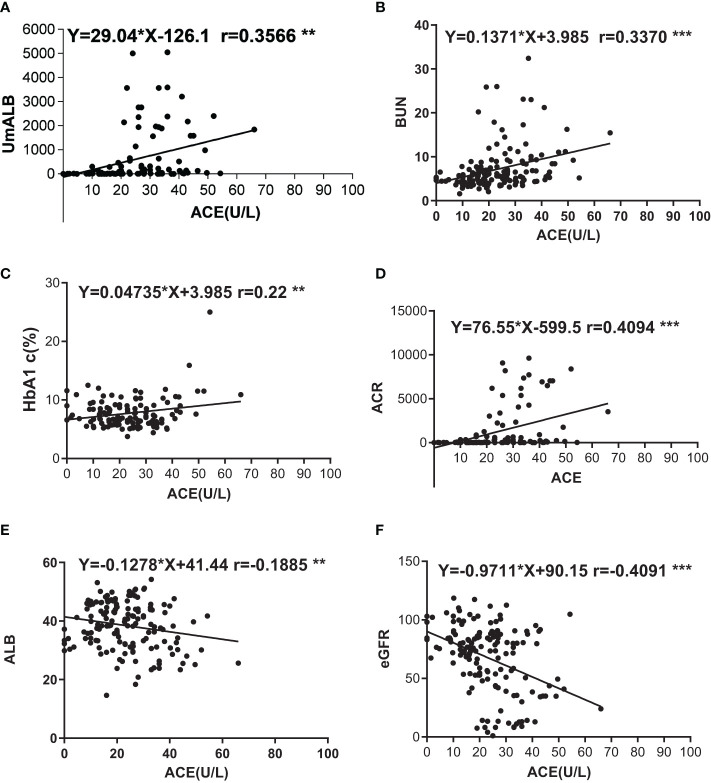
Correlations of ACE with other indexes. **(A)** Correlation between UmALB level and ACE. Y = 29.04x - 126.141.0, R^2^ = 0.121; **(B)** Correlation between BUN level and ACE. Y = 1371x + 3.985, R^2^ = 0.109; **(C)** Correlation between HbA1c level and ACE. Y = 0.04735x + 6.621, R^2^ = 0.050; **(D)** Correlation between ACR level and ACE. Y = 76.55x - 599.5, R^2^ = 0.164; **(E)** Correlation between ALB level and ACE. Y = -0.1278x + 41.44, R^2^ = 0.035; **(F)** Correlation between GFR level and ACE. Y = -9711x + 90.15, R^2^ = 0.160. **P < 0.01; ***P < 0.001.

It has been widely accepted that ACR is a frequently-used laboratory index to diagnose DN and distinguish early-stage (30 mg/g < ACR < 300mg/g) and advanced-stage (ACR > 300 mg/g) DN (31). Based on ACR levels, DN patients were divided into four subgroups of early-stage DN, early-stage DN combined with DR, advanced-stage DN, and advanced-stage DN patients DR. Key clinical and laboratory data of every subgroup were summarized in **Table 4**, and the ACE levels were exhibited in **Figure 1**. Statistical analysis proved that the ACE level in early-stage DN significantly dropped in comparison with the advanced-stage DN or early-stage DN combined with DR (*P* < 0.05) (**Figure 1**).

**Table 4:** Characteristics of different DN subgroup patients (mean ± SD).

Parameter	Early-stage DN	Early-stage DN+DR	Advanced-stage DN	Advanced-stage DN+DR		F	*P* value (two-tailed)
Age (years)	62.88 ± 11.78	62.09 ± 12.19	63.09 ± 10.18	59.00 ± 10.57		0.67	0.571
Gender, M/F, n	7/10	8/3	14/9	19/5		0.12	0.729^*^
BMI (kg/m^2^)	23.29 ± 2.32	22.67 ± 1.75	22.87 ± 3.04	22.95 ± 2.10		0.17	0.917
ALB (g/L)	38.33 ± 6.24	36.44 ± 4.98	32.08 ± 8.78	31.52 ± 6.92		3.98	0.011
ACE (μmol/L)	19.91 ± 11.09	31.03 ± 10.50	29.16 ± 11.95	29.77 ± 9.96		3.63	0.017
UmALB (mg/L)	51.70 ± 49.96	91.45 ± 55.58	985.39 ± 1496.75	867.35 ± 1246.80		10.10	0.000
BUN (μmol/L)	6.31 ± 3.16	6.58 ± 1.93	11.26 ± 7.90	11.20 ± 5.99		3.90	0.012
HbA1c (%)	8.97 ± 1.81	9.37 ± 3.16	7.54 ± 2.34	7.29 ± 1.63		3.35	0.025
Ucr (μmol/L)	5231.18 ± 3005.14	7204.73 ± 3310.38	5524.40 ± 4537.16	5191.17 ± 2934.31		0.99	0.406
eGFR [ml.min^-1^. (1.73m^2^)]	71.31 ± 17.31	68.88 ± 31.47	35.49 ± 30.42	37.01 ± 27.09		9.29	0.000
TB (μmol/L)	9.85 ± 4.48	11.82 ± 5.40	6.62 ± 2.56	6.20 ± 3.36		7.91	0.000
DB (μmol/L)	4.52 ± 1.94	5.38 ± 2.88	3.34 ± 1.38	2.70 ± 1.58		6.80	0.000
TBA (μmol/L)	7.96 ± 14.55	11.19 ± 9.59	5.65 ± 3.13	4.42 ± 2.47		2.02	0.119
TG (mmol/L)	2.23 ± 1.64	5.26 ± 6.16	2.54 ± 3.26	1.97 ± 1.00		3.08	0.033
HDL (mmol/L)	1.57 ± 1.34	1.25 ± 1.06	0.98 ± 0.29	0.97 ± 0.31		2.28	0.088
LDL (mmol/L)	2.98 ± 1.83	3.29 ± 1.04	2.84 ± 1.05	3.19 ± 0.94		0.44	0.727
TC (mmol/L)	4.94 ± 2.63	5.26 ± 2.23	4.58 ± 1.45	4.96 ± 1.30		0.34	0.798
HDL/LDL	0.91 ± 1.43	0.44 ± 0.44	0.33 ± 0.20	0.31 ± 0.14		2.82	0.044
ACR (mg/g)	96.96 ± 78.53	119.55 ± 70.25	1964.36 ± 2822.68	3829.29 ± 3131.94		10.60	0.000

^*^Chi-square test and P < 0.05 was considered statistically significant.

## Discussion

DN and DR is main microvascular complication of T2DM. DN is one of the most important causes of end-stage renal disease (ESRD), and DR would evolve into blind, at present, DN and DR rapidly increasing to be a popular disease in China (32). In clinical practice, DN is characterized with proteinuria; however, diet, sports, and other factors could affect the levels of proteinuria (33). Searching a biomarker to distinctively recognize DN to replace UmALB is helpful and valuable to diagnose DN. In our research, we aimed to assess the variation of ACE levels in DN patients compared with T2DM and healthy and the value of ACE levels to diagnose DN. Our results manifested that ACE level in the DN patients was significantly higher than that in the control or in the T2DM which may partly demonstrate that ACE levels may be a new marker to inflect DN. When we divided DN patients with ACR and considered whether DN patients companied with DR to subgroup, further subgroup analysis results showed that early-stage DN patients develop into advanced-stage or early-stage DN patients companied with DR, and serum ACE levels obviously increased. Above results of our study exhibited that elevated serum ACE levels may be a valuable biomarker to discern DN progression and early-stage DN patients with impaired retina.

Diabetic vascular complications are responsible for most of the mortality and morbidity in diabetic populations worldwide (34). The complications are divided into macrovascular and microvascular. Macrovascular complications include coronary artery disease and cerebrovascular disease, and microvascular complications cover diabetic retinopathy (DR) and DN (35). DR may have the identical pathologic change as DN; when T2DM patients have impaired retina, it is thought to be an indicator of DN (36). In T2DM patients, DR and proteinuria could be decisive for the decline of renal function (37). There is evidence that when T2DM patient was diagnosed with DR, it would be essential to assess their kidney function and DR may predict the renal outcomes of DN patients (38). In our study, DR and DN coexisted in 35 patients, where the ACE level in these patients was higher than that in another 40 DN patients without DR; in particular, when ACR was <300 mg/g, ACE level in patients with DN concomitant DR was the time is prominently higher than that in DN patients without DR. It is reasonable to predict that ACE level could elevate in T2DM patients accompanied by retinopathy. Our study revealed that ACE level in DN patients was significantly higher than that in the control or T2DM; further subgroup analysis confirmed obvious elevation of ACE level in advanced-stage DN in comparison with the early-stage DN, and when early-stage DN patients companied with DR, serum ACE levels will also increase than early-stage DN patients.

As we all know that, RAAS plays vital roles to maintain plasma sodium, arterial blood pressure, and extracellular volume homeostasis. Angiotensinogen, angiotensin I, angiotensin II, and angiotensin converting enzyme are all important composition of RAAS, and Ang I cleaved into Ang II by ACE at lung capillaries, endothelial cells, and kidney epithelial cells (39–41). A study also reported that AngIIof intra-renal was 50–100 times higher than the circulatory AngII (42). Proteinuria is a renal pathology and also the clinical characteristic of DN; however, high RAAS activity will cause or aggravate albuminuria (43). Higher level of ACE reflects vast AngII, and a mass of AngIIindicates high RAAS activity. We imagined that higher serum ACE levels in DN patients were strongly correlated with proteinuria, and we probed into the correlation between serum ACE levels and renal function indexes such as BUN, UmALB, ACR, and eGFR, as you could see in **Supplementary Figure S2**. Strong relevance was gotten between ACE and UmALB (r = 0.3566), BUN (r = 0.337), ACR (r = 0.4094), and eGFR (r = -0.4091). The consequences also prove the idea that ACE is a potential biomarker to diagnose DN.

The present study has potential limitations. Firstly, as it was conducted in single Chinese population, ethic group difference should be regarded when popularizing the conclusion to other ethnics. Secondly, the number of patients recruited in this study was relatively small; therefore, enlarged sample size is required for further confirmation of the results.

In summary, our results imply that elevated serum ACE levels in DN patients may be an indicator for diabetic nephropathy, and continuously increased ACE is a possible signal of diabetic nephropathy progression. Additionally, increased ACE levels may be a single of retina impaired of early-stage DN patients.

## Data Availability Statement

The raw data supporting the conclusions of this article will be made available by the authors, without undue reservation.

## Ethics Statement

The studies involving human participants were reviewed and approved by ethics committee of Xiangya Hospital of Central South University. The patients/participants provided their written informed consent to participate in this study.

## Author Contributions

All authors participated in the design, interpretation of the studies, analysis of the data, and review of the manuscript. KH designed and analyzed the data and drafted the manuscript. YL and KW conducted the experiment. JW, HL, and YM collected the data. Bin Yi supplied critical reagents and reviewed and edited the text.

## Conflict of Interest

The authors declare that the research was conducted in the absence of any commercial or financial relationships that could be construed as a potential conflict of interest.

## Publisher’s Note

All claims expressed in this article are solely those of the authors and do not necessarily represent those of their affiliated organizations, or those of the publisher, the editors and the reviewers. Any product that may be evaluated in this article, or claim that may be made by its manufacturer, is not guaranteed or endorsed by the publisher.
