# Supplementary figures and images for: Elevated ACE Levels Indicate Diabetic Nephropathy Progression or Companied Retina Impaired
Source: Front Clin Diabetes Healthc. 2022 May 12;3:831128. doi: 10.3389/fcdhc.2022.831128 (PMC10012155; doi:10.3389/fcdhc.2022.831128)

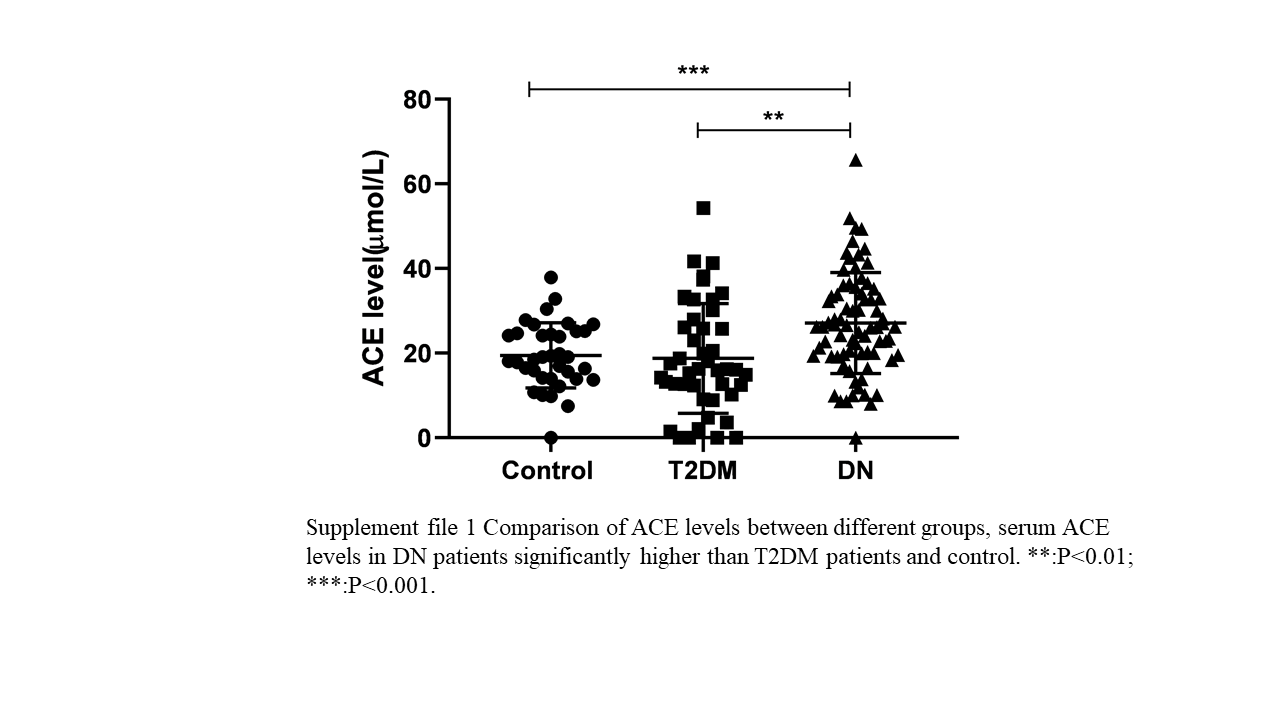

Supplement: Supplementary file 1 [file Image_1.tif]

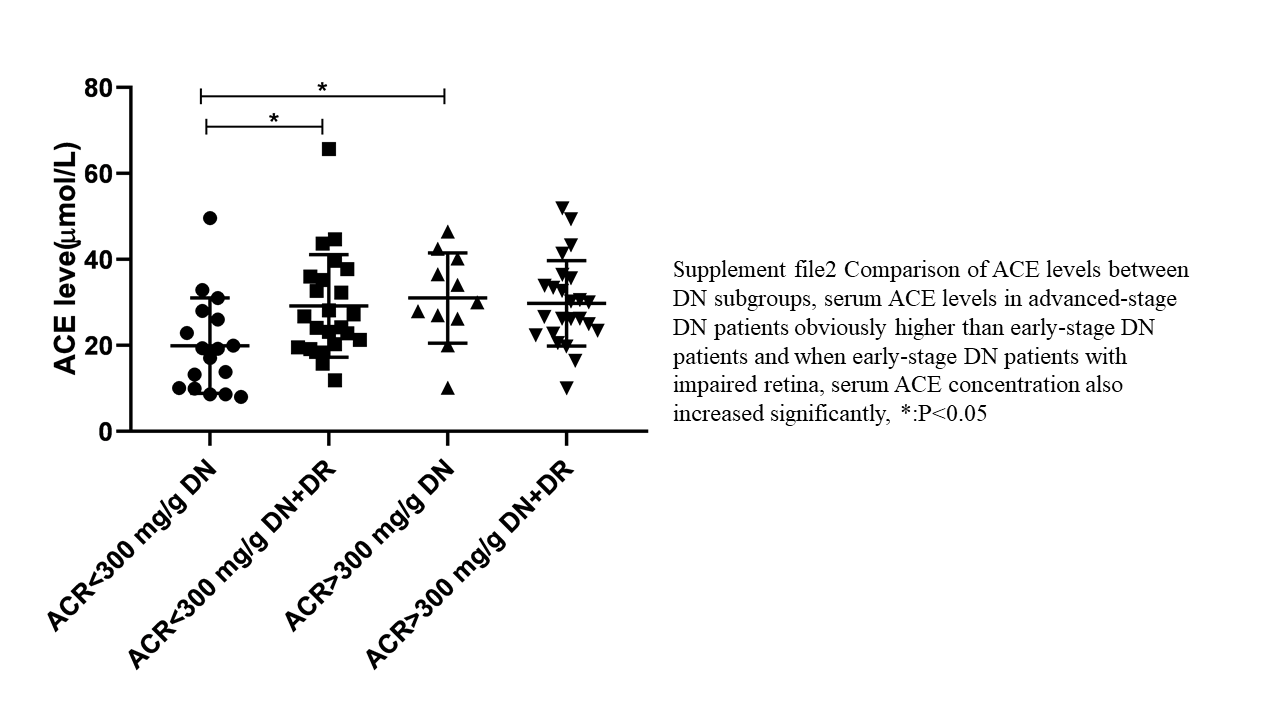

Supplement: Supplementary file 2 [file Image_2.tif]
